# Sirolimus-Coated Balloon Angioplasty of Infra-popliteal Lesions for the Treatment of Chronic Limb-Threatening Ischemia: Study Protocol for the Randomized Controlled LIMES Study

**DOI:** 10.1007/s00270-022-03213-z

**Published:** 2022-07-29

**Authors:** Ulf Teichgräber, Stephanie Platzer, Thomas Lehmann, Maja Ingwersen, René Aschenbach, Ulrich Beschorner, Dierk Scheinert, Thomas Zeller

**Affiliations:** 1 Department of Radiology, Jena University Hospital, Friedrich-Schiller-University Jena, Am Klinikum 1, 07747 Jena, Germany; 2 Center for Clinical Studies, Jena University Hospital, Friedrich-Schiller-University Jena, Jena, Germany; 3 Department of Angiology, University Heart Center Freiburg-Bad Krozingen, Bad Krozingen, Germany; 4 Department of Angiology, University Hospital Leipzig, Leipzig, Germany

**Keywords:** Balloon angioplasty, Peripheral artery disease, Sirolimus

## Abstract

**Purpose:**

Evidence on efficacy and long-term safety of paclitaxel-coated devices is still conflicting. Therefore, this study aims to assess whether sirolimus-coated balloon angioplasty is safe and effective for the treatment of infra-popliteal occlusions in patients with chronic limb-threatening ischemia (CLTI).

**Study design:**

The randomized controlled, single-blinded, multicentre, investigator-initiated study aims to enrol 230 participants with CLTI and infra-popliteal occlusions at up to 25 centres. Patients will be randomized in a 1:1 ratio to either sirolimus-coated balloon angioplasty or to plain old balloon angioplasty (POBA). Bailout stenting in case of flow-limiting dissection or ≥ 50% residual diameter stenosis is permitted.

**Outcome measures:**

Primary outcome is the Kaplan–Meier estimate of primary patency at 6 months, defined as the absence of target lesion occlusion with restoration of in-line flow to the ankle. Key secondary outcome is non-inferiority in the proportionate occurrence of major adverse limb events and perioperative all-cause death at 30 days. Overall, participants will be followed for 36 months to assess further secondary efficacy and safety outcomes.

**Assumed gain of knowledge:**

If sirolimus-coated balloon angioplasty turns out to be superior to uncoated-balloon angioplasty regarding patency of infra-popliteal lesions without safety signals, it could become a welcome treatment option for patients with CLTI.

*Trial Registration* ClinicalTrial.gov Identifier: NCT04772300, German Clinical Trials Register: DRKS00024629.

*Level of Evidence* Level 2a, randomized controlled trial.

## Introduction

Lower-extremity peripheral arterial disease (PAD) affects an estimated 27 million adults in Europe and North America. Main risk factors are smoking, diabetes, dyslipidaemia, and hypertension. The prevalence of PAD rises with age up to 20% among those over 70 years [1]. In advanced PAD, presenting as chronic limb-threatening ischemia (CLTI) the risk of limb loss and cardiovascular death is substantially increased, and thus, current guidelines recommend urgent revascularization [2–4].

Until now, plain old balloon angioplasty (POBA) and provisional bare-metal stenting (BMS) are the standard care for infra-popliteal endovascular revascularization. However, efficacy is not satisfactory. Drug-eluting stents (DESs) have shown to decrease the risk of restenosis in short lesions [5]; however, until now, there is no evidence that this also applies for longer lesions. In addition, permanent implants are associated with potential disadvantages including in-stent restenosis and stent thrombosis. Recent studies found considerable evidence of vessel wall degeneration with paclitaxel-eluting stents [6]. In contrast, angioplasty with drug-coated balloon (DCB) leaves no implant behind and drug release is briefer. However, superiority of paclitaxel-coated balloon angioplasty over POBA is still contradictory [5, 7–14]. Furthermore, long-term safety of paclitaxel-coated devices is still controversial because of the cytotoxic property of paclitaxel and the risk of distal embolization [15–20]. Whether sirolimus, which is characterized by a wider therapeutic range, may serve as a safe and effective alternative to paclitaxel remains to be proven.

The objective of our multicentre, randomized controlled trial is to evaluate whether infra-popliteal angioplasty in patients with CLTI with the commercially marketed MAGIC Touch PTA sirolimus-coated balloon catheter (Concept Medical Inc., Tampa, FL, USA) is superior to POBA regarding efficacy and non-inferior to POBA regarding safety.

## Study Design

**Fig. 1:**
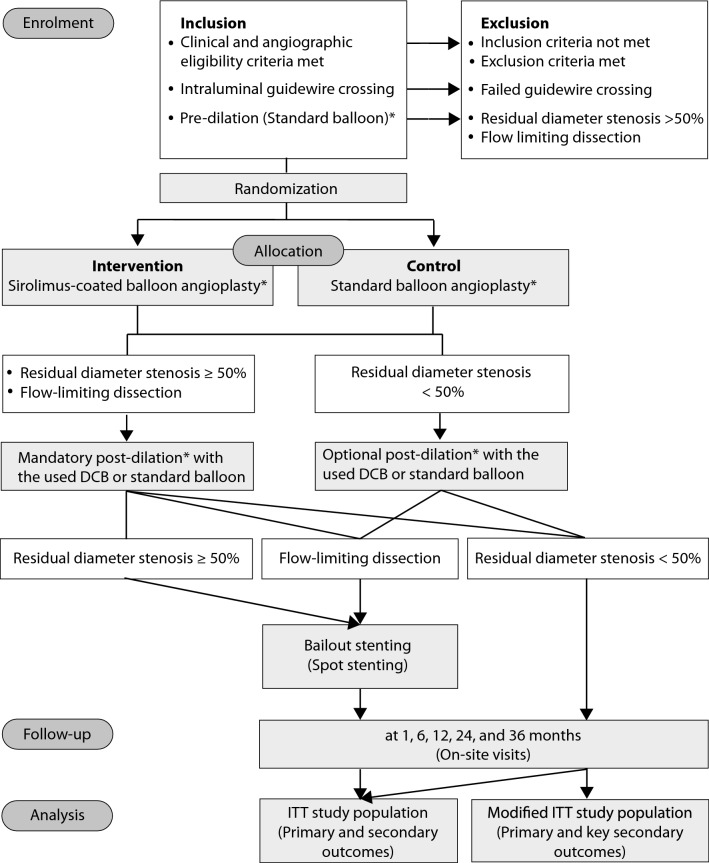
Study flow chart. *Pre-dilation, drug-coated balloon dilation, standard balloon dilation, and post-dilation should last for at least 60 s each. Dilation for 180 s each is strongly recommended. *DCB* drug-coated balloon, *ITT* intention to treat

The LIMES study (Prospective Multi-Centre Randomized Controlled Trial to Evaluate the Safety and Efficacy of Sirolimus Drug Coated Versus Non-Coated Standard Balloon Angioplasty for the Treatment of Infra-popliteal Occlusions in Patients with Peripheral Arterial Disease) is a prospective, multicentre, single-blinded, investigator-initiated, randomized controlled trial. Up to 25 centres are intended to participate. Eligible patients who provided written informed consent will be allocated at a ratio of 1:1 to either DCB angioplasty with the study device (MAGIC Touch PTA sirolimus-coated balloon catheter) or to POBA with a marketed uncoated balloon catheter (Fig. 1). All study devices have the European certificate of conformity (CE mark). Participants and the independent core laboratory, but not the investigators will be blinded to the treatment allocation. The Friedrich-Schiller-University ethics committee, Jena, Germany approved the study. In addition, written permissions from the respective local ethics committees will be obtained by all participating sites. The study is registered with ClinicalTrials.gov (NCT04772300) and with the German clinical trials register (DRKS00024629). The first patient was enrolled on March 4, 2022.

## Eligibility Criteria

### Inclusion Criteria

Individuals are eligible for trial participation if the following criteria applied.

1. Age ≥ 18 years.
2. Clinical symptoms meet Rutherford category 4 to 6.
3. In case of Rutherford category 5 or 6: individuals with infection grade 0–2 and/or ischemia grade 2–3 according to the Wound, Ischemia, and foot Infection (WIfI) classification.
4. Infra-popliteal lesion above the ankle joint (lesions must not extend above the tibioperoneal trunk or below the tibiotalar joint; treatment must not extend beyond these boundaries for more than 1 cm). A target lesion may extend into the distal popliteal artery segment in case it involves a straight uninterrupted lesion extending from the target vessel. Non-significant stenosis below the ankle joint can be allowed if it is not part of the target lesion and does not require treatment.
5. Participants can only be enrolled once with a single target lesion.
6. Total occlusion (de novo or not stent related reocclusion) of the target vessel as assessed by angiography.
7. The target lesion may consist of multiple stenoses/occlusions of a single target vessel if they are ≤ 5 cm apart and if at least one total occlusion occurs.
8. In case of multiple occluded infrapopliteal arteries, the investigator should assign, at his discretion, that artery as target vessel that is expected to contribute most to the restoration of the in-line flow to the ankle. Non-target vessels can be treated during the same procedure according to the patient’s treatment allocation.
9. Reference vessel diameter (RVD) ≥ 2 mm and ≤ 4 mm by angiographic visual estimate
10. Patency of inflow arteries (< 50% diameter stenosis). Inflow stenosis may be treated during the index procedure to ensure sufficient inflow. Treatment must achieve ≤ 30% residual diameter stenosis. There must be a minimum of 3 cm of healthy vessel between the treated inflow lesion and the target lesion. Use of paclitaxel-coated devices is not permitted.
11. Intraluminal guidewire crossing
12. Successful pre-dilation of the target lesion (≤ 50% residual diameter stenosis and no flow-limiting dissection ≥ Grad D according to the National Heart, Lung, and Blood Institute classification)
13. Participant’s declaration of informed consent

### Exclusion Criteria

Individuals are excluded from trial participation if any of the following criteria applied.

1. Stroke or heart attack less than 30 days prior to enrolment.
2. History of or planned target limb amputation above the metatarsal level.
3. Neurotrophic ulcers, heel pressure ulcers, or calcaneal ulcers with risk of major amputation.
4. Any vascular surgical procedure or intervention performed in the target limb within 30 days prior to or planned within 30-day post-index procedure.
5. Stent implantation or bypass surgery in the target vessel prior to randomization (if a stent is already present in the target vessel, there must be a distance of at least 3 cm to the target lesion).
6. Vascular treatment with drug-coated balloons within 6 months prior to index procedure.
7. Known or suspected active infection other than associated with target limb wounds.
8. Infection grade 3 and ischemia grade 0 and/or 1 according to WIfI classification.
9. Active osteomyelitis beyond cortical involvement of the bone (excluding phalanges).
10. Vasculitis, systemic lupus erythematosus, or polymyalgia rheumatica on active treatment.
11. Presence of fresh thrombus in the target lesion.
12. Presence of aneurysm in the target vessel.
13. Systemic corticosteroid therapy (expected dosage > 5 mg of prednisolone or equivalent per day within 9 months after the index procedure).
14. Known allergies or sensitivity to heparin, aspirin, other anticoagulant/antiplatelet therapies, sirolimus, paclitaxel, or contrast media that cannot be adequately pre-treated prior to index procedure.
15. Target lesion requires treatment with alternative therapies such as primary stenting, laser, lithotripsy, thrombectomy, atherectomy, brachytherapy, re-entry devices (intra-arterial lysis ≥ 4 weeks before the index procedure is permitted).
16. Significant gastrointestinal bleeding or any coagulopathy that would contraindicate anti-platelet therapy.
17. Pregnant or lactating women
18. Life expectancy of less than one year in the opinion of the investigator.
19. Participant is enrolled in another investigational drug-, device-, or biologic study.

## Intervention

After intraluminal guide wire crossing, pre-dilation of the target lesion with a standard balloon catheter for at least 60 s (180 s strongly recommended) is mandatory in all participants. Nominal diameter should match the distal reference vessel diameter. Immediately after successful pre-dilation, participants will be randomly allocated to either angioplasty with sirolimus-coated balloon (s) (intervention group) or angioplasty with uncoated standard balloon (s) (control group). Participants but not investigators will be blinded to the allocation. Prior to the study intervention, inflow-stenosis ≥ 50% must be treated to achieve ≤ 30% diameter stenosis.

Balloon dilation for angioplasty of the target lesion with either DCB or POBA should be maintained for at least 60 s. Maximum inflation time as per institution’s standard of care is recommended. Nominal balloon diameter should match the distal reference vessel diameter. Balloon length should exceed the target lesion by at least 5 mm proximal and 5 mm distal. If more than one balloon is necessary for complete lesion coverage, multiple balloons should overlap by at least 5 mm. In participants with ≥ 50% residual diameter stenosis or flow-limiting dissection, a prolonged post-dilation of at least 60 s (180 s strongly recommended) must be performed. In case of < 50% residual diameter stenosis, post-dilation is left to the investigator’s discretion. If post-dilation remains unsuccessful (residual diameter stenosis ≥ 50% or flow-limiting dissection), bailout stenting with self-expanding bare nitinol stent (s) is permitted (Fig. 1).

### Concomitant Medication

Antiplatelet therapy should be used in both study arms according to clinical routine and under consideration of concomitant diseases. Prior to the index procedure, it is strongly recommended to administer dual antiplatelet therapy (DAPT) as a combination of aspirin (100 mg daily at least 3 days before the procedure or a loading dose of 500 mg) and clopidogrel (75 mg daily at least 3 days before the procedure or a loading dose of 300 mg), or aspirin and rivaroxaban (2.5 mg twice a day) per hospital standard of care. During the index procedure, participants must receive appropriate anticoagulation by means of heparin according to the institution’s standard of care. DAPT is recommended for at least 4 weeks after index procedure (aspirin 100 mg daily, clopidogrel 75 mg daily) and single antiplatelet therapy indefinitely thereafter (aspirin 100 mg daily) according to centres’ standard of care.

## Outcome Measures

The participant timeline is presented in Table 1.

**Table 1:** Schedule of enrolment, intervention, and assessment

Study period	Enrolment		Allocation	Angioplasty	Post-Procedure	Follow-up				
		Index procedure								
Timepoint	*− t*_1_*	*− t*_2_†	0	Immediately after allocation	At discharge§	1 month ± 7 days On-site	6 months ± 30 days On-site	12 months ± 45 days On-site	24 months ± 60 days On-site	36 months ± 60 days On-site
ENROLMENT										
Eligibility screen	X									
Informed consent	X									
Allocation			X							
INTERVENTION										
Intraluminal guide wire crossing^‡^		X								
Pre-dilation**		X								
Sirolimus-coated balloon angioplasty				X						
Standard balloon angioplasty				X						
ASSESSMENT										
Demographic data	X									
Physical examination^††^	X				X	X	X	X	X	X
Laboratory examination^‡‡^	X									
Medical history	X									
Concomitant medication	X				X	X	X	X	X	X
Angiography		X		X						
Duplex ultrasonography^§§^					X	X	X	X	X	X
ABI	X				X	X	X	X	X	X
TBI	X				X	X	X	X	X	X
Rutherford category	X					X	X	X	X	X
Wound evaluation	X				X	X	X	X	X	X
Walking distance (Participant self-assessment***)	X					X	X	X	X	X
VascuQuol Score	X					X	X	X	X	X
EQ-5D-3L index	X					X	X	X	X	X
AE/SAE		X		X	X	X	X	X	X	X

ABI, ankle-brachial index; AE, adverse event; EQ-5D-3L index, European quality of life 5 dimensions 3 level index; SAE, severe adverse event; TBI, toe-brachial index

^*^At baseline

^†^During index procedure

^‡^Successful intraluminal guidewire crossing of the lesion

^§^Within two working days after index procedure and before discharge

^**^Pre-dilation with residual diameter stenosis < 50% (by angiographic visual estimate) and without flow limiting dissection

^††^Including blood pressure and heart rate

^‡‡^Creatinine, platelets, white blood cell count, pregnancy test were appropriate

^§§^Duplex ultrasonography should also be performed before and after any target vessel revascularization. Adjudication by core laboratory at 6, 12, and 24 months

***Participant self-assessment of maximum walking distance should be performed before completion of the questionnaires

### Primary Outcome

Primary efficacy outcome is the composite of limb salvage and primary patency at 6 months (Kaplan–Meier estimate). Primary patency is defined as the absence of target lesion occlusion with restoration of in-line flow to the ankle [3] without repeat clinically driven target lesion revascularization (TLR). TLR is considered clinically driven in case of ≥ 70% diameter stenosis (by quantitative angiography conducted by the core laboratory) and deterioration of Rutherford category and/or deterioration or persistence of wounds according to the WIfI classification wound component score.

Primary patency as assessed with duplex ultrasonography (DUS) must be confirmed by the independent, blinded core laboratory. DUS images and medical records will be made available for review by the clinical events committee upon request.

### Secondary Outcomes

*Key secondary safety outcome* is the composite of major adverse limb events and perioperative all-cause death (MALE-POD) at 30 days (non-inferiority). MALE is defined as above the ankle amputation or major intervention (new bypass graft, interposition graft revision, or thrombectomy/thrombolysis) of the target limb that involves infra-popliteal arteries.

### Further Secondary Outcomes

- Device success (deployment of the study device according to manufacturer’s instructions of use).
- Technical success (device success and absence of target lesion occlusion with restoration of in-line flow to the ankle).
- Procedural success (technical success and absence of major adverse events (MALE-POD, myocardial infarction, stroke) within 72 h of the index procedure) [21]
- Composite of limb salvage and primary patency at 1, 12, 24, and 36 months
- Clinically driven TLR/target vessel revascularization (TVR) at 1, 6, 12, 24, and 36 months
- Primary/secondary target lesion patency at 1, 6, 12, 24, and 36 months
- Target lesion reocclusion at 1, 6, 12, 24, and 36 months
- Amputation-free survival at 1, 6, 12, 24, and 36 months
- All-cause death at 1, 6, 12, 24, and 36 months
- Change in Rutherford classification at 1, 6, 12, 24, and 36 months
- Change in ankle-/toe-brachial index at 1, 6, 12, 24, and 36 months
- Primary/secondary clinical improvement at 1, 6, 12, 24, and 36 months
- Change in EQ-5D-3L index/VascuQuol Score at 1, 6, 12, 24, and 36 months
- Change in self-reported walking distance at 1, 6, 12, 24, and 36 months
- Wound healing at 1, 6, 12, 24, and 36 months
- Days of hospitalization at 1, 6, 12, 24, and 36 months

Angiographic imaging and DUS at 6, 12, and 24 months will be adjudicated by a blinded core laboratory.

### Statistics

Based on results from the Lutonix BTK trial and the ACOART-BTK study [13, 22], we assumed a 55% rate of primary patency in the control group. Given a minimally clinical important difference of 20% (benefit from the study intervention) to be detected with 80% probability at a two-sided 5% level of significance and considering 20% of drop-out, 230 participants (115 in each group) need to be recruited. As incidences of 30-day freedom from MALE-POD in the Lutonix BTK trial were about 99% in both groups, 230 participants are also sufficient to provide 90% power to prove non-inferiority of sirolimus-coated balloon angioplasty to POBA regarding the key secondary safety endpoint at a one-sided 2.5% significance level. The primary efficacy endpoint will be assessed with Kaplan–Meier analysis applying the log-rank test. The key secondary safety endpoint will be assessed with the Farrington–Manning test. The non-inferiority margin is sat at 10%. Further secondary endpoints will be compared using the two-sided t-test, the Mann–Whitney *U* test, and the log-rank test where appropriate. Subgroup analysis will be conducted to consider diabetes, lesion length, and distal run-off. Analyses will be based on data from the intention-to-treat (ITT) study population (enrolled participants, irrespective of whether or not they underwent the allocated intervention). Primary outcome and key secondary outcome will additionally be analysed based on the modified ITT study population (participants who received the assigned treatment).

## Discussion

The randomized controlled LIMES study was designed to evaluate efficacy and safety of the MAGIC Touch PTA sirolimus-coated balloon catheter for the treatment of infra-popliteal arteries in patients with CLTI. Long-lasting patency and sufficient limb perfusion are essential for limb salvage and survival.

Although the combination of balloon angioplasty and local antiproliferative therapy appears promising, the efficacy of paclitaxel-coated balloons for the treatment of infra-popliteal lesions is still conflicting [9, 13, 14]. In addition, concerns about long-term safety of paclitaxel-coated devices are not dispelled so far. Thus, since challenges related to the coating technology are mastered, sirolimus is suspected to turn out as safe and effective alternative to paclitaxel. Sirolimus is a cytostatic, but not, as paclitaxel, cytotoxic drug. It is characterized by a wide therapeutic window. Phospholipid carriers ensure an improved adhesion to the balloon surface to prevent drug loss into the blood stream. The excipient should ensure an effective transfer of sirolimus into deeper vessel layers where it retains for as long as necessary to inhibit neointima formation [23].

The LIMES study will provide data on patency, clinical improvement, wound healing, health-related quality of life, recurrent revascularization, amputation, and mortality throughout 36 months.

Sirolimus could be suitable to effectively support angioplasty without reducing safety or impede possible later surgical intervention. If sirolimus-coated balloon angioplasty will prove to be effective regarding patency and non-inferior to POBA regarding safety, it may replace POBA where needed and serve as an alternative to paclitaxel-coated balloon angioplasty.

## Abbreviations

ABI: Ankle-brachial index
BMS: Bare metal stent
CLTI: Chronic limb-threatening ischemia
DAPT: Dual antiplatelet therapy
DCB: Drug-coated balloon
DES: Drug-eluting stent
DUS: Duplex ultrasonography
ITT: Intention to treat
MALE-POD: Major adverse limb events and perioperative death
PAD: Peripheral artery disease
POBA: Plain old balloon angioplasty
TBI: Toe-brachial index
TLR: Target lesion revascularization
TVR: Target vessel revascularization
WIfI: Wound, Ischemia, and foot Infection classification
